# Asparaginase Potentiates Glucocorticoid-Induced Osteonecrosis in a Mouse Model

**DOI:** 10.1371/journal.pone.0151433

**Published:** 2016-03-11

**Authors:** Chengcheng Liu, Laura J. Janke, Jitesh D. Kawedia, Laura B. Ramsey, Xiangjun Cai, Leonard A. Mattano, Kelli L. Boyd, Amy J. Funk, Mary V. Relling

**Affiliations:** 1 Department of Pharmaceutical Sciences, St. Jude Children’s Research Hospital, Memphis, Tennessee, United States of America; 2 Department of Pathology, St. Jude Children’s Research Hospital, Memphis, Tennessee, United States of America; 3 Department of Pharmacy Research, The University of Texas MD Anderson Cancer Center, Houston, Texas, United States of America; 4 Department of Pediatrics, Cincinnati Children’s Hospital Medical Center, Cincinnati, Ohio, United States of America; 5 HARP Pharma Consulting, Mystic, Connecticut, United States of America; 6 Department of Pathology Microbiology and Immunology, Vanderbilt University Medical Center, Nashville, Tennessee, United States of America; 7 Animal Resource Center (Veterinary Services), St. Jude Children’s Research Hospital, Memphis, Tennessee, United States of America; University of Ulm, GERMANY

## Abstract

Osteonecrosis is a common dose-limiting toxicity of glucocorticoids. Data from clinical trials suggest that other medications can increase the risk of glucocorticoid-induced osteonecrosis. Here we utilized a mouse model to study the effect of asparaginase treatment on dexamethasone-induced osteonecrosis. Mice receiving asparaginase along with dexamethasone had a higher rate of osteonecrosis than those receiving only dexamethasone after 6 weeks of treatment (44% vs. 10%, P = 0.006). Similarly, epiphyseal arteriopathy, which we have shown to be an initiating event for osteonecrosis, was observed in 58% of mice receiving asparaginase and dexamethasone compared to 17% of mice receiving dexamethasone only (P = 0.007). As in the clinic, greater exposure to asparaginase was associated with greater plasma exposure to dexamethasone (P = 0.0001). This model also recapitulated other clinical risk factors for osteonecrosis, including age at start of treatment, and association with the systemic exposure to dexamethasone (P = 0.027) and asparaginase (P = 0.036). We conclude that asparaginase can potentiate the osteonecrotic effect of glucocorticoids.

## Introduction

Osteonecrosis is a common complication in patients treated for acute lymphoblastic leukemia (ALL), primarily due to the use of glucocorticoids (e.g. dexamethasone and prednisone) [1–5]. Development of osteonecrosis has been associated with impaired blood supply and subsequent bone death. In patients, it commonly affects hips and knees, and can result in pain, limited range of motion and debilitation, often requiring surgical intervention. Proposed mechanisms of glucocorticoid-induced osteonecrosis include inhibition of angiogenesis, bone marrow adipogenesis, hypercoagulation, and apoptosis of endothelial cells and osteocytes.[6] Our study recently suggested that damage to blood vessels supplying bone (arteriopathy) plays a primary role in the pathogenesis of osteonecrosis.[7] Clinical studies have identified several risk factors for osteonecrosis in ALL patients, including adolescent age,[1–5, 8–12] female sex,[1, 2, 4, 8, 12] white race,[2, 10] and exposure to intensive, long-term glucocorticoids.[1–5]

The reported frequency of osteonecrosis has varied widely from 1% to 20% among different ALL protocols [1–5, 8–12] even with relatively similar glucocorticoid regimens, and interactions with accompanying antileukemic medication is suspected to contribute to this variability. Asparaginase is a critical component of ALL regimens and is often given concurrently with steroids. Evidence is accumulating that asparaginase can increase the risk of osteonecrosis. Asparaginase exposure was associated with decreased clearance and increased exposure of dexamethasone,[13] and the development of an antibody response against asparaginase is associated with a decreased incidence of symptomatic osteonecrosis.[14] Recent ALL trials showed that additional doses of asparaginase during interim maintenance therapy caused more osteonecrosis in patients receiving prednisone.[15, 16] Moreover, the hypoalbuminemia caused by asparaginase was also associated with osteonecrosis risk.[3]

However, these clinical data are not definitive, in that they do not come from a trial in which patients receive identical glucocorticoid regimens with different exposures to asparaginase. Thus, our study aimed to address the impact of asparaginase on osteonecrosis in a controlled, pre-clinical model. We also evaluated the impact of host factors (e.g. age and gender), treatment factors (e.g. glucocorticoid regimen and duration), and environmental factors (mice derived from in-house breeding colonies vs. shipped from vendor) on dexamethasone tolerance and the development of osteonecrosis using this mouse model.

## Materials and Methods

### Chemicals

Dexamethasone sodium phosphate solution was purchased from American Pharmaceutical Partners, Inc. (Schaumburg, IL). Pegylated *E*.*coli*-asparaginase (PEG-asparaginase) was a gift from Sigma Tau (Gaithersburg, MD). Tetracycline and bovine serum albumin was purchased from Sigma-Aldrich (St. Louis, MO), and sulfamethoxazole/trimethoprim oral suspension was obtained from Hi-Tech Pharmacal Co., Inc. (Amityville, NY).

### Animals

Unless otherwise specified, studies used male BALB/cJ mice (age 24 or 28 days) bred in-house (St. Jude Children’s Research Hospital, Memphis, TN; the original breeding pairs were from Jackson Laboratories). Several experiments included BALB/cJ mice directly shipped from Jackson Laboratories (Bar Harbor, ME), or male BALB/cAnNHsd mice (age 24 or 28 days) obtained from Harlan Laboratories (Houston, TX). An irradiated folic-acid deficient diet (purchased from TestDiet, Richmond, IN) containing less than 0.05 ppm folic acid was used [17]. Prior to the initiation of all experiments, both in-house bred and vendor-derived mice were transferred into a dedicated experimental room where they were maintained in sterile micro-isolator cages (Micro Vent System 75 JAG, Allentown, NJ) and housed on ventilated racks, with up to 5 mice in a cage with corncob bedding (Andersons Bed-O’cobs, Pharmaserv, Framingham, MA). The mice had access to food and water ad libitum.

### Ethics statement

This study was carried out in strict accordance with the recommendations in the Guide for the Care and Use of Laboratory Animals of the National Institutes of Health. Mice were housed in an American Association of Laboratory Animal Care-accredited facility and were treated using Institutional Animal Care and Use (IACUC)-approved protocols (Protocol Number: 423–100257) in accordance with National Institutes of Health guidelines. Health checks were done twice a day at a minimum and those animals that became moribund or lost 20% of their maximum body weights were immediately euthanized according to IACUC-approved procedures.

### Asparaginase and dexamethasone pharmacokinetics (PK)

**Fig 1:**
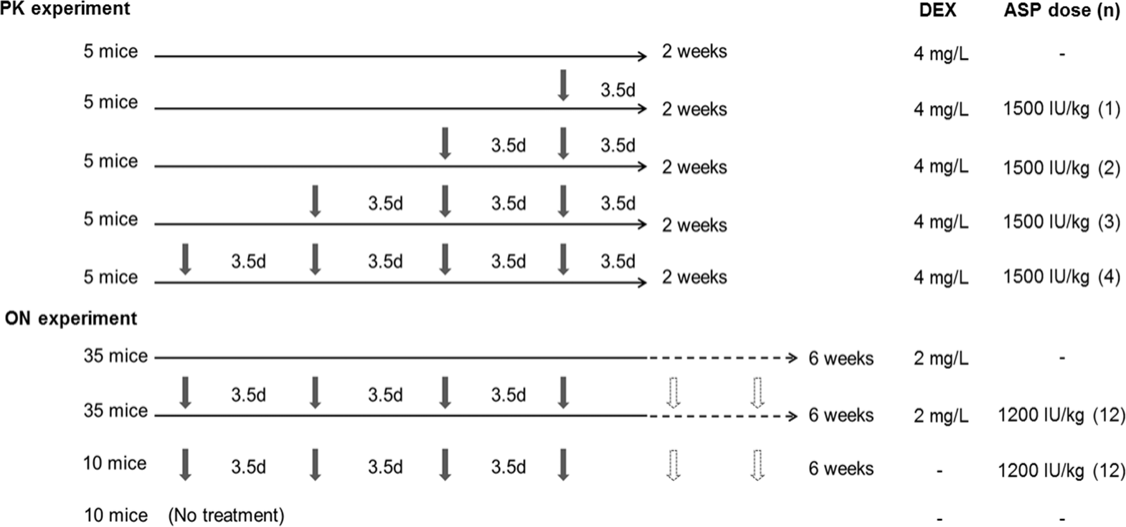
Dexamethasone and asparaginase treatment regimens. Horizontal arrows represent dexamethasone treatment (2 or 4 mg/L in drinking water) and vertical arrows represent PEG-asparaginase (Oncaspar) treatment (1200 or 1500 IU/kg twice weekly via i.p. injection). Dashed lines and arrows indicate continuous dexamethasone and twice weekly asparaginase treatment after week 2. The dose of dexamethasone in drinking water and dose and number of asparaginase injections are shown.

The effect of asparaginase treatment on dexamethasone PK, was studied in 6–8 week old male mice. Dexamethasone was given in the drinking water at 4 mg/L and PEG-asparaginase (Oncaspar) at 1,500 IU/kg intraperitoneally (i.p.) for 0 to 4 doses at an interval of 3.5 days (Fig 1). All mice were sacrificed at the end of week-2 (3.5 days after last asparaginase dose).

### Mouse model of dexamethasone-induced osteonecrosis

In our previous study,[17] 14 strains of mice for their susceptibility of osteonecrosis were screened on the basis of their known constitutive phenotypes which might predispose to osteonecrosis. BALB/c, a strain with high platelet counts, was shown to have the highest rate of osteonecrosis after receiving dexamethasone in the drinking water. The increased susceptibility in this strain may be due to high platelets and poor collateral circulation in the hind limb,[18] because arteriopathy and subsequently impaired blood supply have been indicated as the primary cause of osteonecrosis.[7] Dexamethasone was more strongly associated with osteonecrosis in clinical leukemia trials than is prednisone,[19] and therefore used in our murine model.

A series of experiments were conducted to refine this model (S1 Methods). From postnatal day 28 (P28) or day 24 (P24), male and female BALB/cJ and BALB/cAnN mice were treated with 4 or 8 mg/L dexamethasone in the drinking water, equivalent to 1.33 or 2.66 mg/kg/day, assuming that each mouse weighed 15 g and consumed 5 mL water daily.[20] Mice in dexamethasone groups were treated with dexamethasone with antibiotics, except in a pilot experiment where mice were given dexamethasone without antibiotics. The control groups were treated with only antibiotics. Treatment generally lasted for 6 weeks, while a subset of BALB/cAnN was treated for up to 8 weeks in an effort to increase the frequency of osteonecrosis. Antibiotic prophylaxis to prevent dexamethasone-induced infections consisted of tetracycline (1 g/L) continually, and sulfamethoxazole (600 mg/L) and trimethoprim (120 mg/L) given 3.5 days per week. These antibiotics have no effect on osteonecrosis.[17] Water bottles were changed every 3.5 days.

### Effect of asparaginase on osteonecrosis

Male P24 BALB/cJ in-house bred mice were used to determine the effect of asparaginase on dexamethasone-induced osteonecrosis. Ninety mice were divided into four groups: 35 mice were assigned to the dexamethasone-alone group, 35 mice to the dexamethasone and asparaginase group, 10 mice to the asparaginase-alone group and 10 mice to untreated control (Fig 1). Because prolonged treatment of dexamethasone (4 mg/L in the drinking water for 6 weeks) and asparaginase (1,500 IU/kg twice a week) resulted in high mortality (~60%; data not shown) of 4-week-old BALB/c mice due to sepsis, we reduced the dose of dexamethasone to 2 mg/L and PEG-asparaginase to 1,200 IU/kg i.p. twice a week. The same antibiotic prophylaxis was used as described above.

### Plasma dexamethasone concentration and asparaginase activity

At the time of sacrifice (between 9am and noon), mice were anesthetized with 2% isoflurane, and blood was collected via cardiac puncture. Plasma was frozen at -80°C until assayed. Dexamethasone concentration was quantified by HPLC.[21] Plasma asparaginase activity was determined using a high-throughput assay by monitoring the enzymatically-coupled oxidation of reduced nicotinamide adenine dinucleotide (NADH) to NAD(+).[22] The linear range of the assay was established from 0.025 to 2.2 IU/mL; if the activity was out of the range, the plasma was diluted with 5% bovine serum albumin to be in range.

### Detection of osteonecrosis and arteriopathy

Osteonecrosis was determined by histological evaluation as described previously.[7, 17, 20] At the time of sacrifice, both hind limbs were collected, fixed in 10% formalin overnight, decalcified in TBD2 (Thermo Fisher Scientific, Waltham, MA), paraffin-embedded, sagittally sectioned, stained with hematoxylin and eosin, and evaluated for the presence of osteonecrosis in the distal femur. We focused on the distal femur because it was the only joint affected in our previous study.[17] Osteonecrosis was diagnosed if all the following were present: empty lacunae, pyknotic nuclei or ghost nuclei in osteocytes in the bone trabeculae, and necrosis of the adjacent marrow and stromal elements. Arteriopathy was defined by the presence or absence of lesions in arteriolar branches of the medial genicular artery located along the surface of the distal femoral condyles.[7] If there was no evaluable arteriolar branch present, the case was categorized as “unknown”. Mice with osteonecrosis and/or arteriopathy in one or both legs were classified as positive for osteonecrosis and/or arteriopathy.

### Statistical analysis

The χ^2^ test was used to evaluate intergroup differences in categorical variables. The Mann-Whitney test was used to compare continuous variables. A P-value of less than 0.05 was considered statistically significant.

## Results

### Plasma dexamethasone concentration was increased by asparaginase treatment

**Fig 2:**
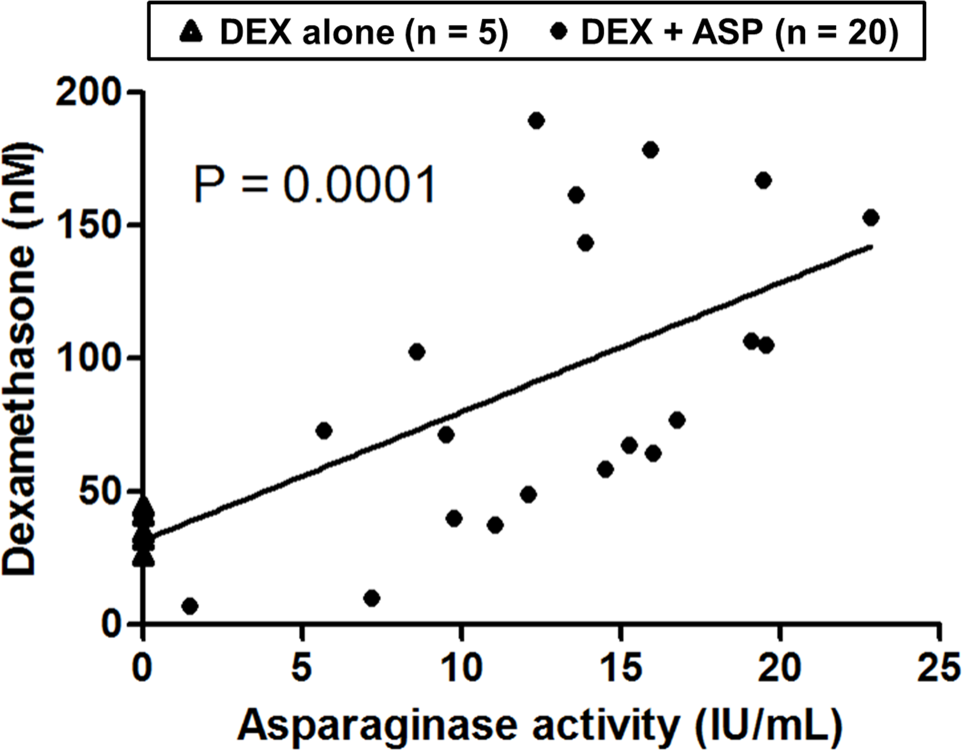
Plasma dexamethasone concentration was positively associated with asparaginase activity in PK experiment. Mice received dexamethasone (DEX; 4 mg/L in drinking water) for 2 weeks and 0–4 doses of PEG-asparaginase (ASP; 1500 IU/kg i.p.) at 3.5 day intervals. Blood samples from dexamethasone-alone mice (triangles) and those received additional asparaginase (points) were collected at the end of week 2 (3.5 days after the last asparaginase injection). Linear regression line is shown. PK, pharmacokinetics.

After 2 weeks of treatment with dexamethasone at 4 mg/L and PEG-asparaginase at 1,500 IU/kg for 0–4 doses, all 25 mice in the PK experiment were evaluable at the end of week 2. There was no significant difference in plasma asparaginase activity among the three groups (15.3 ± 6.5, 14.5 ± 7.7 and 13.2 ± 4.1 IU/mL, respectively) that received 2, 3 or 4 doses of asparaginase at 1,500 IU/kg i.p. every 3.5 days. Those who received only one dose of asparaginase had slightly lower asparaginase activity (11.9 ± 2.6 IU/mL, P = 0.02) compared with other groups. We observed a positive association between plasma dexamethasone concentration and asparaginase activity (P = 0.0001; Fig 2). No mice developed osteonecrosis after only two weeks of treatment.

### Asparaginase treatment potentiated osteonecrosis and arteriopathy in dexamethasone-treated mice

Based on the result of preliminary experiments, we studied the effect of asparaginase on dexamethasone-induced osteonecrosis using 24-day-old BALB/cJ male mice as described in Fig 1. The mean weights of mice treated with asparaginase (14.7 ± 1.9 g with dexamethasone and 19.4 ± 2.5 g without dexamethasone) were significantly lower than those who did not receive asparaginase (17.4 ± 1.5 g with dexamethasone and 26.8 ± 2.3 g without dexamethasone; P < 0.0001) after 6 weeks of treatment. Thirty of 35 (86%) mice in the dexamethasone-alone group and 27 of 35 (77%; P = 0.8) mice in the dexamethasone plus asparaginase group survived the 6-week treatment. There were no deaths among the 10 untreated controls and the 10 asparaginase-alone mice.

**Fig 3:**
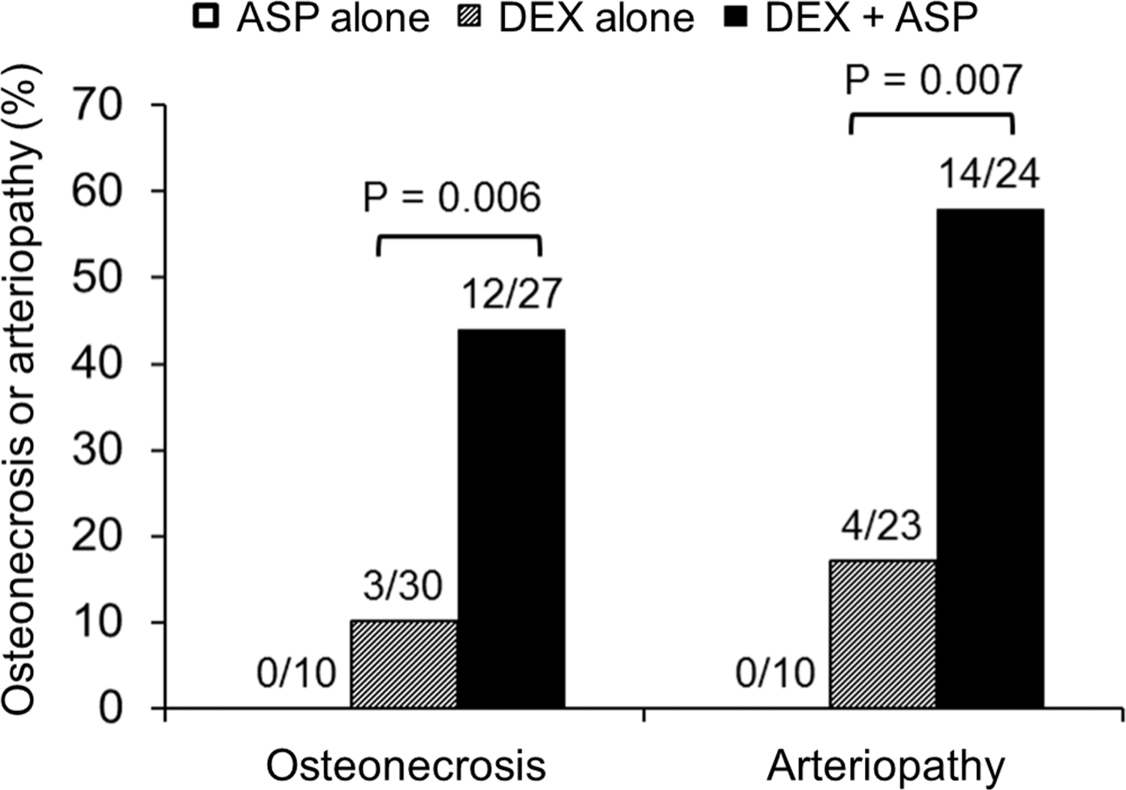
Asparaginase treatment potentiated osteonecrosis and arteriopathy in dexamethasone-treated mice. Chi-square P values were calculated between dexamethasone (DEX)-treated mice that received vs. those that did not receive asparaginase (ASP).

Osteonecrosis developed in 12 of 27 (44.4%) mice who received dexamethasone and asparaginase, which is significantly more frequent (P = 0.006) than in mice who received dexamethasone alone (3 of 30 or 10.0%; Fig 3). An arteriolar branch coursing along the dorsal surface of the distal femur was detected in 47 of the 57 mice evaluated for osteonecrosis. Lesions were present in the vessels of 14 of 24 (58%) mice who received both dexamethasone and asparaginase, compared with 4 of 23 (17%) mice who received dexamethasone alone (P = 0.007). No mice in the control group or the asparaginase alone group developed either osteonecrosis or arteriopathy.

**Fig 4:**
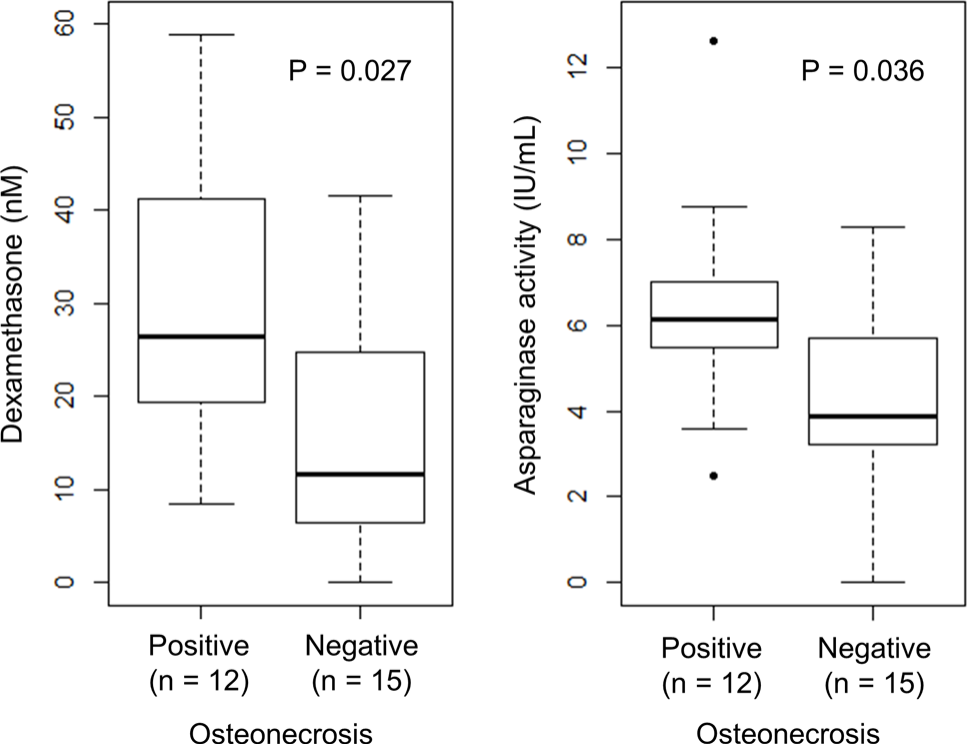
Osteonecrosis was associated with higher plasma dexamethasone and asparaginase levels in mice receiving both dexamethasone and asparaginase treatment. BALB/cJ males received dexamethasone (4 mg/L) and PEG-asparaginase (1200 IU/kg i.p. twice weekly) for 6 weeks. Blood samples were collected at the end of week 6 (approximately 3.5 days after last asparaginase injection).

Consistent with the pharmacokinetic experiment, we observed an association between plasma dexamethasone concentration and asparaginase activity (P = 0.005; S1 Fig) at the end of 6 weeks in the osteonecrosis experiment, In the 27 mice that received both dexamethasone and asparaginase, there was a significant trend towards higher dexamethasone levels (P = 0.027; Fig 4A) and higher asparaginase activity (P = 0.036; Fig 4B) in osteonecrosis-positive cases. A multivariate analysis including plasma dexamethasone and asparaginase levels suggested that dexamethasone (OR = 1.01 for every 1 nmol/L increase, P = 0.029) and asparaginase concentrations (OR = 1.06 for every 1 IU/mL increase, P = 0.002) were both independently associated with osteonecrosis.

We previously showed preliminary data that native asparaginase could potentiate glucocorticoid-induced osteonecrosis.[17] However, native *E*.*coli*-asparaginase is no longer commercially available; it has been replaced by PEG-asparaginase. In most ALL protocols, PEG-asparaginase is administered intravenously or intramuscularly, and the typical doses range from 1000 IU/m^2^ to 3500 IU/m^2^.[22–25] Plasma asparaginase activity at 3 days after receiving a usual recommend dose of PEG-asparaginase (2500 IU/m^2^ i.v.) ranged from 2 to 4 IU/mL in patients.[26] Herein, we used doses of PEG-asparaginase (1200–1500 IU/kg i.p.) that have previously been shown to result in antileukemic effects in murine models.[27] After injection of PEG-asparaginase at 1200 IU/kg in mice, we achieved plasma asparaginase activity that was slightly higher (5.4 ± 2.9 IU/mL) than those in patients receiving a standard dose (2500 IU/m^2^), and may be more comparable to patients on high-dose asparaginase therapies.

### Arteriopathy was likely the initiating event of osteonecrosis

Among the 15 mice positive for osteonecrosis, one did not have an evaluable vessel present in the plane of section, and all the other 14 (100%) were positive for arteriopathy. Among the 33 mice with no signs of osteonecrosis, arteriopathy was present in 4 mice (12%; P = 9 × 10^−9^), consistent with our previous study which indicated that vascular damage may be the initiating event of osteonecrosis development.[7]

**Fig 5:**
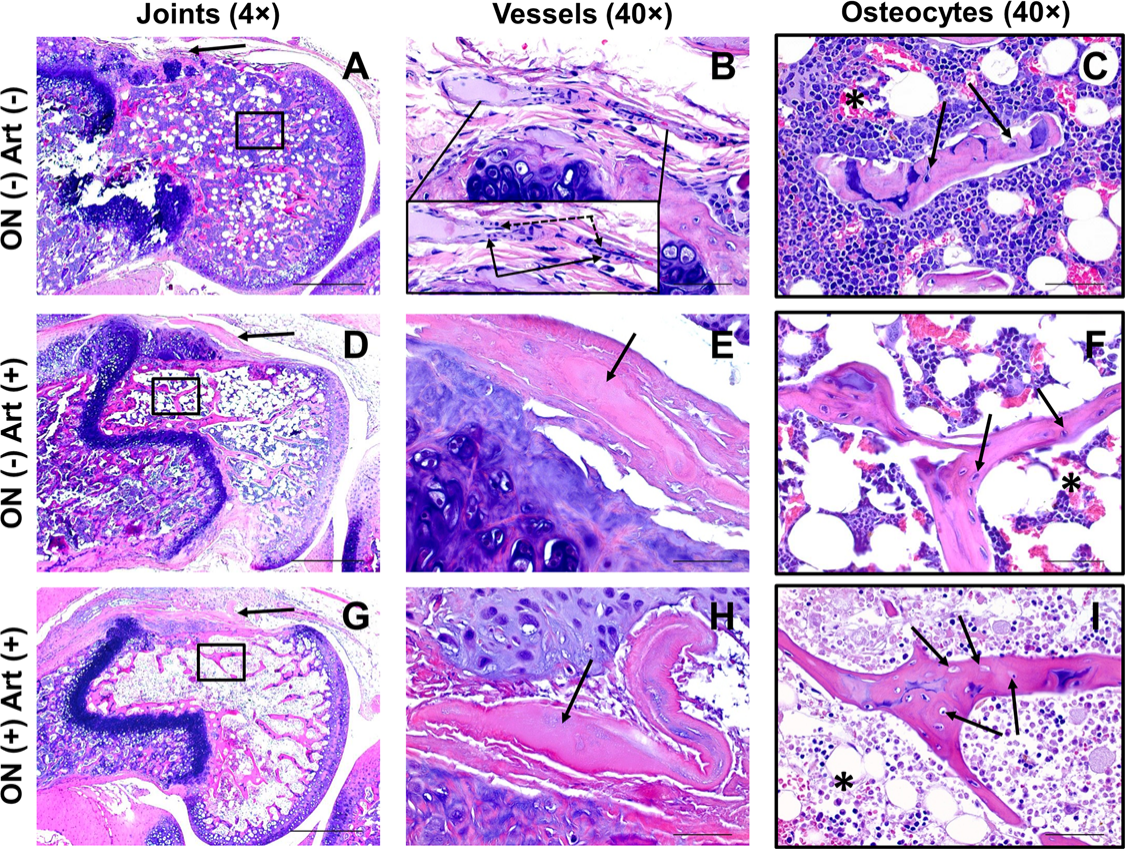
Histology of osteonecrosis and arteriopathy. H&E staining of representative stifle joints from mice negative for osteonecrosis (ON) and arteriopathy (Art, top panel), negative for osteonecrosis but positive for arteriopathy (middle panel), and positive for both osteonecrosis and arteriopathy (bottom panel). All mice received dexamethasone and PEG-asparaginase for 6 weeks. (A) Normal arteriole (arrow), marrow and trabecular bone. (B) Magnified cross-section of the arteriole in A. The inset shows the normal endothelial cells (dashed arrows) with elongated nuclei and smooth muscle cells (solid arrows) with round nuclei. (C) Magnification of boxed area in A, showing healthy osteocytes in lacunae (arrows) and healthy hematopoietic cells in marrow (asterisk). (D) Vessel with arteriopathy (arrow) and healthy bone with slightly decreased hematopoietic cells (asterisk). (E) Magnification of the arteriole in D, showing thickened, occluded blood vessel (arrow) and loss of endothelium and smooth muscle cells. (F) Magnification of boxed area in D, showing healthy osteocytes in lacunae (arrows) and decreased hematopoietic cells (asterisk). (G) Vessel with arteriopathy (arrow) and necrotic marrow and trabecular bone. (H) Magnification of the arteriole in G, showing thickened, occluded blood vessel (arrow) and loss of endothelium and smooth muscle cells. (I) Magnification of boxed area in G, showing empty lacunae and dead osteocytes (arrows) and necrotic marrow (asterisk). Scale bars are 500 microns in the left panel and 50 microns in the middle and right panels.

The histopathological changes of bone and vessels during dexamethasone and asparaginase treatment are shown in Fig 5. When arteriopathy was present without osteonecrosis (Fig 5D), it was evident in branches of the medial genicular artery supplying the distal femoral epiphysis (Fig 5E) with signs of luminal occlusion and loss of endothelium and smooth muscle cells; the interruption of blood supply was localized, resulting in reduced hematopoietic cells in bone marrow (Fig 5F). When arteriopathy and osteonecrosis were both present, long-term poor circulation led to death of osteocytes and necrosis of marrow and hematopoietic cells (Fig 5I) and ultimately, extensive necrosis of the bone (Fig 5G).

## Discussion

Clinical studies have indicated an interaction between asparaginase and glucocorticoids in pediatric patients receiving these two critical agents of ALL chemotherapy.[13, 28] Herein, we used a mouse model to confirm the clinical observations that systemic exposure of glucocorticoids was increased by asparaginase treatment, and we showed definitively that asparaginase treatment contributes to the osteonecrotic effect of glucocorticoids.

In the present study, we confirmed the positive association between plasma dexamethasone level and asparaginase activity in two independent experiments (Fig 2 and S1 Fig). Concurrent use of asparaginase and glucocorticoids can potentiate each other’s effects. Asparaginase is associated with decreased clearance of dexamethasone,[13] which could be due to its hypoproteinemic effect, possibly decreasing hepatic CYP3A or transporters. Also, the immunosuppressive effects of glucocorticoids inhibit the antibody response against asparaginase and prevents its neutralizing effect, which in turn results in higher plasma asparaginase activity.[14] In a front-line ALL study, St. Jude Total XV, patients with antibodies against asparaginase had a lower risk of developing osteonecrosis than those who did not develop antibodies.[14]

Clinically, asparaginase is always used in therapy which includes glucocorticoids. Hanada et al[29] reported a pediatric ALL patient who developed osteonecrosis during asparaginase therapy, but the patient had also received prednisone. In the present study, we did not observe any significant change in bone or vessels of mice receiving asparaginase alone, consistent with asparaginase enhancing the osteonecrotic effect of glucocorticoids, rather than a direct impact on osteonecrosis.

There are several possible mechanisms for the potentiating effect of asparaginase on glucocorticoid-induced osteonecrosis. Both asparaginase and glucocorticoids have been shown to induce a hypercoagulable state by suppression of anticoagulant factors such as antithrombin, plasminogen and d-dimer, and by elevation in F VIII/vWF complex.[30] The hypercoagulable state may lead to impaired circulation, vascular damage and subsequent osteonecrosis.[29, 31–33] Interestingly, patients between 11 and 16 years had more significant alteration of anticoagulant and fibrinolytic parameters than children of other ages,[34] consistent with the high susceptibility of adolescents to osteonecrosis. Moreover, alterations in lipid metabolism after asparaginase treatment [35, 36] may lead to formation of lipidic droplets that can be entrapped in the arterial lumen, followed by reduced blood flow and damage to the vascular endothelium. Asparaginase treatment is also associated with venous stasis and deep vein thrombosis in clinic [37] and in animal models,[38] although we did not observe such effects with asparaginase alone.

Despite the anatomical and genetic differences between mouse and human, this mouse model yielded similar lesions in bone and arteries as those that have been reported clinically. Biopsy specimens of the femoral head from patients with early-stage osteonecrosis showed structural damage to arteriolar walls before necrosis of trabecular bone and marrow.[39] We tested multiple host-related and treatment-related risk factors for osteonecrosis using this model (details shown in S1 Methods). Osteonecrosis was associated with male sex, earlier start of treatment (before the onset of puberty), and higher dose and longer treatment duration with dexamethasone. All of these predisposing factors have been reported in clinical studies;[2–4, 8–10, 12] however, in clinical studies that do show a gender difference, females are at higher risk than males.[2, 4, 8, 40, 41] Why male mice have a higher risk than females is not clear. Among the mice of different substrains, sources and ages, four-week-old in-house bred BALB/cJ male mice displayed high susceptibility to osteonecrosis with an acceptable survival rate; therefore they may serve as a reliable and reproducible mouse model for studying glucocorticoid-induced osteonecrosis in the future.

Almost all contemporary front-line ALL regimens contain glucocorticoids and asparaginase during induction therapy. The interaction between these two important antileukemic drugs can cause inter-individual variability in dexamethasone and asparaginase pharmacokinetics, which may influence the efficacy and toxicity of ALL treatment. This animal model allows us to study the optimal dosing strategy of glucocorticoids and asparaginase, and explore the utility of new biomarkers for osteonecrosis.

In summary, we were able to recapitulate the potentiating effect of asparaginase on glucocorticoid-induced osteonecrosis using a mouse model. Mice receiving asparaginase concomitantly with dexamethasone developed more arteriopathy and subsequent osteonecrosis than those received dexamethasone alone. The drug interaction should be considered when using glucocorticoids and asparaginase concomitantly in the treatment of ALL.

## Supporting Information

S1 Fig Plasma dexamethasone concentration was positively associated with asparaginase activity in osteonecrosis experiment. Mice received dexamethasone (DEX; 4 mg/L in drinking water) for 6 weeks (triangles) or dexamethasone and PEG-asparaginase (ASP; 1200 IU/kg i.p.) at 3.5 day intervals (12 doses in total) for 6 weeks (points). Samples were collected at the end of week 6. Linear regression line is shown.
(DOCX)

S2 Fig Survival differed by sources of mice and treatment regimens. Left: Kaplan-Meier curves of vendor-derived and in-house bred BALB/cJmales treated with different dexamethasone regimens beginning on postnatal day 28. Mice were given either 4 mg/L throughout the 6-week treatment period (low-dose), or 8 mg/L for the first week and 4 mg/L thereafter (high-dose). Note that all mice except a small group (n = 4) received prophylactic antimicrobials to prevent infection. Right: frequency of osteonecrosis by groups. Chi-square P value = 0.025 for the comparison between in-house bred and vendor-derived mice on the same regimen.
(DOCX)

S3 Fig Plasma concentrations of dexamethasone. Dexamethasone concentrations measured at the time of euthanasia in BALB/cJ males (vendor-derived vs in-house bred) treated with dexamethasone (DEX) administered at 4 mg/L.
(DOCX)

S4 Fig Gender-dependent differences in susceptibility to osteonecrosis. (A) Kaplan-Meier curve of in-house bred BALB/cJ males and females treated with dexamethasone at 4 mg/L. (B) Frequency of osteonecrosis in male and female mice treated with dexamethasone at 4 mg/L for 4–6 weeks. (C) Plasma dexamethasone concentration in in-house bred BALB/cJ males and females treated with dexamethasone at 4 mg/L.
(DOCX)

S1 Methods Mouse model of dexamethasone-induced osteonecrosis (with references). (DOCX)
