# Supplementary material for: Asparaginase Potentiates Glucocorticoid-Induced Osteonecrosis in a Mouse Model
Source: PLoS One. 2016 Mar 11;11(3):e0151433. doi: 10.1371/journal.pone.0151433 (PMC4788417; doi:10.1371/journal.pone.0151433)
Supplement: S1 Fig — Mice received dexamethasone (DEX; 4 mg/L in drinking water) for 6 weeks (triangles) or dexamethasone and PEG-asparaginase (ASP; 1200 IU/kg i.p.) at 3.5 day intervals (12 doses in total) for 6 weeks (points). Samples were collected at the end of week 6. Linear regression line is shown. (DOCX) [file pone.0151433.s001.docx]

**S1 Fig. Plasma dexamethasone concentration was positively associated with asparaginase activity in osteonecrosis experiment.** Mice received dexamethasone (DEX; 4 mg/L in drinking water) for 6 weeks (triangles) or dexamethasone and PEG-asparaginase (ASP; 1200 IU/kg i.p.) at 3.5 day intervals (12 doses in total) for 6 weeks (points). Samples were collected at the end of week 6. Linear regression line is shown.

**
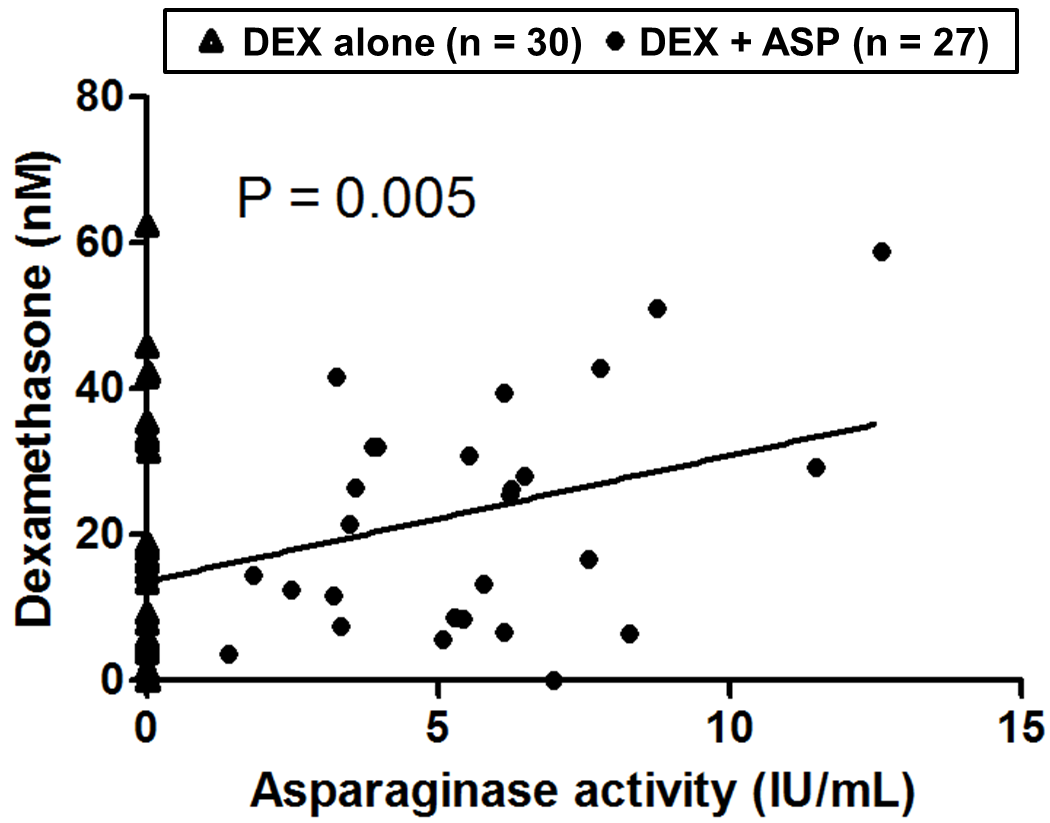
**
