# Supplementary material for: Asparaginase Potentiates Glucocorticoid-Induced Osteonecrosis in a Mouse Model
Source: PLoS One. 2016 Mar 11;11(3):e0151433. doi: 10.1371/journal.pone.0151433 (PMC4788417; doi:10.1371/journal.pone.0151433)
Supplement: S2 Fig — Left: Kaplan-Meier curves of vendor-derived and in-house bred BALB/cJmales treated with different dexamethasone regimens beginning on postnatal day 28. Mice were given either 4 mg/L throughout the 6-week treatment period (low-dose), or 8 mg/L for the first week and 4 mg/L thereafter (high-dose). Note that all mice except a small group (n = 4) received prophylactic antimicrobials to prevent infection. Right: frequency of osteonecrosis by groups. Chi-square P value = 0.025 for the comparison between in-house bred and vendor-derived mice on the same regimen. (DOCX) [file pone.0151433.s002.docx]

**S2 Fig. Survival differed by sources of mice and treatment regimens.** Left: Kaplan-Meier curves of vendor-derived and in-house bred BALB/cJmales treated with different dexamethasone regimens beginning on postnatal day 28. Mice were given either 4 mg/L throughout the 6-week treatment period (low-dose), or 8 mg/L for the first week and 4 mg/L thereafter (high-dose). Note that all mice except a small group (n = 4) received prophylactic antimicrobials to prevent infection. Right: frequency of osteonecrosis by groups. Chi-square P value = 0.025 for the comparison between in-house bred and vendor-derived mice on the same regimen (details in S1 Methods).

**
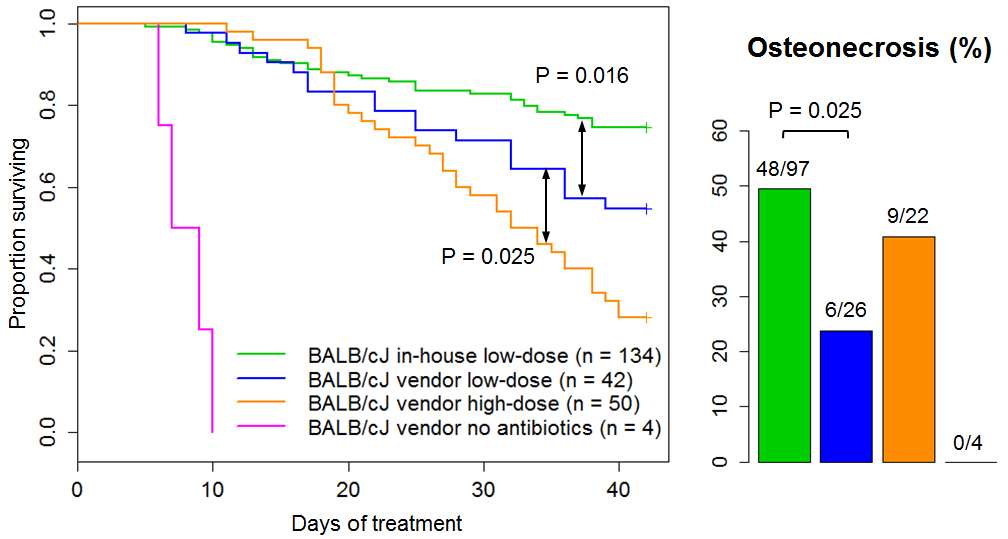
**
