# Supplementary material for: Asparaginase Potentiates Glucocorticoid-Induced Osteonecrosis in a Mouse Model
Source: PLoS One. 2016 Mar 11;11(3):e0151433. doi: 10.1371/journal.pone.0151433 (PMC4788417; doi:10.1371/journal.pone.0151433)
Supplement: S3 Fig — Dexamethasone concentrations measured at the time of euthanasia in BALB/cJ males (vendor-derived vs in-house bred) treated with dexamethasone (DEX) administered at 4 mg/L. (DOCX) [file pone.0151433.s003.docx]

**S3 Fig. Plasma concentrations of dexamethasone.** Dexamethasone concentrations measured at the time of euthanasia in BALB/cJ males (vendor-derived vs in-house bred) treated with dexamethasone (DEX) administered at 4 mg/L (Details in S1 Methods).

**
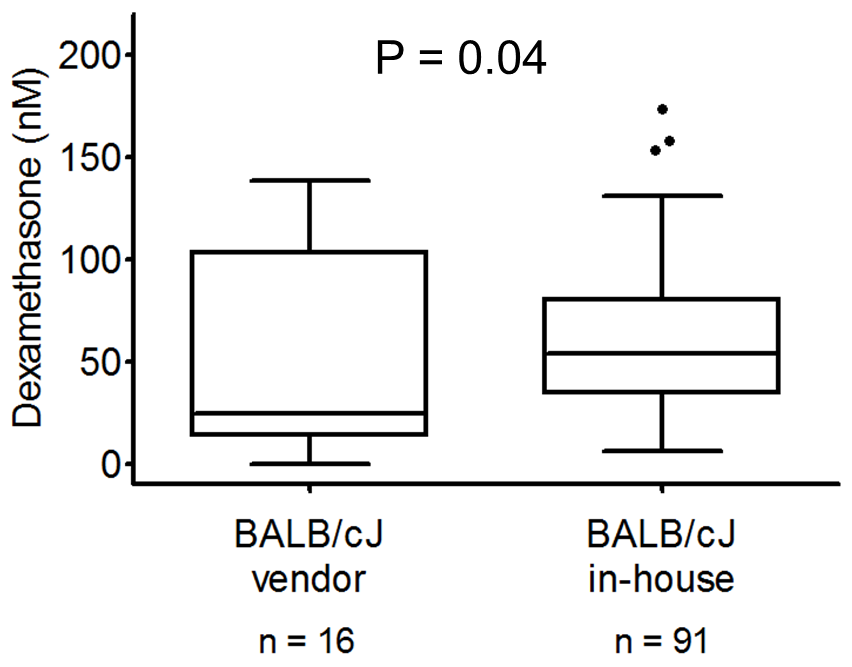
**
