# Supplementary material for: Asparaginase Potentiates Glucocorticoid-Induced Osteonecrosis in a Mouse Model
Source: PLoS One. 2016 Mar 11;11(3):e0151433. doi: 10.1371/journal.pone.0151433 (PMC4788417; doi:10.1371/journal.pone.0151433)

**S4 Fig.** **Gender-dependent differences in susceptibility to osteonecrosis.** (A) Kaplan-Meier curve of in-house bred BALB/cJ males and females treated with dexamethasone at 4 mg/L. (B) Frequency of osteonecrosis in male and female mice treated with dexamethasone at 4 mg/L for 4-6 weeks. (C) Plasma dexamethasone concentration in in-house bred BALB/cJ males and females treated with dexamethasone at 4 mg/L (details in S1 Methods).


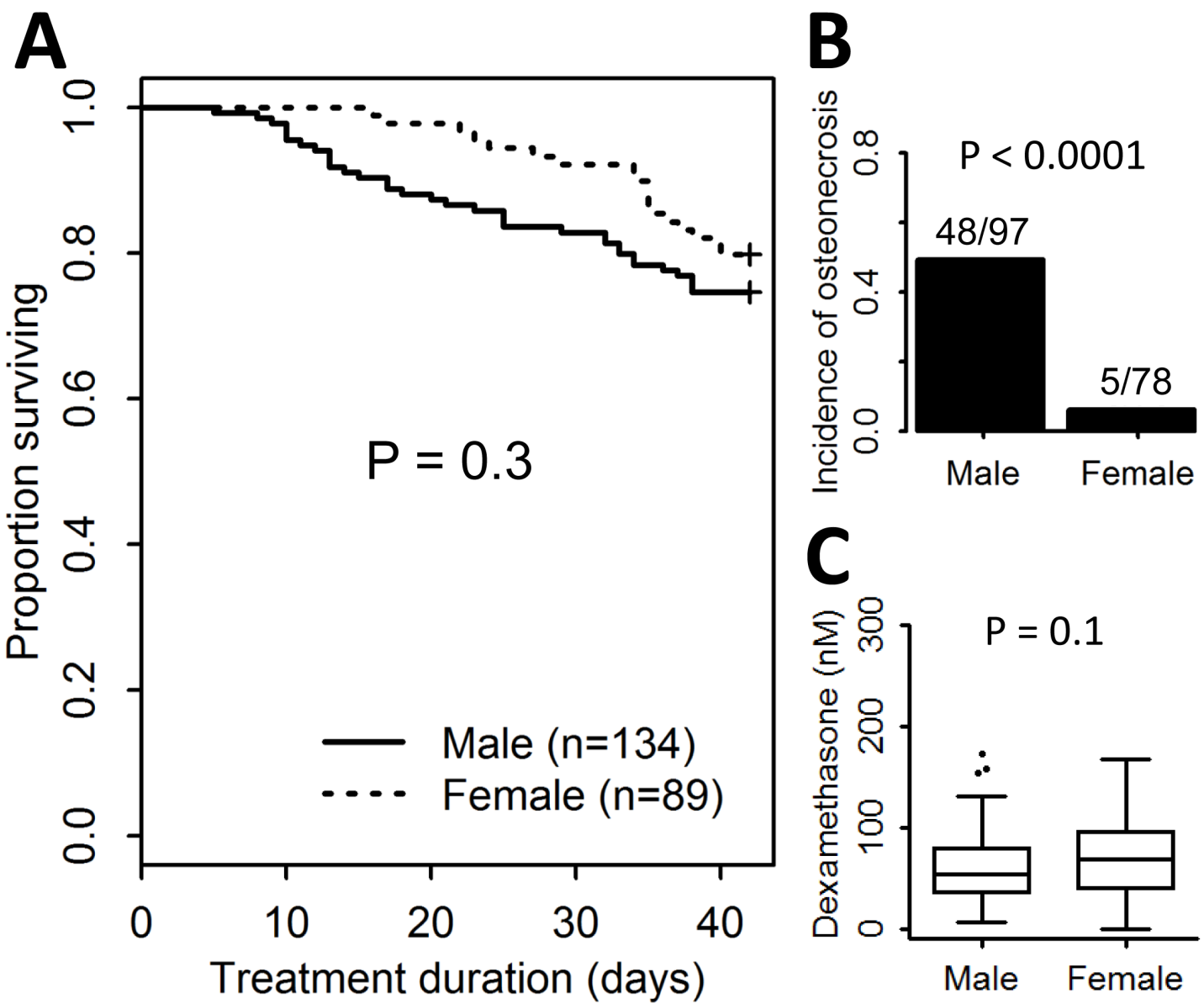

Supplement: S4 Fig — (A) Kaplan-Meier curve of in-house bred BALB/cJ males and females treated with dexamethasone at 4 mg/L. (B) Frequency of osteonecrosis in male and female mice treated with dexamethasone at 4 mg/L for 4–6 weeks. (C) Plasma dexamethasone concentration in in-house bred BALB/cJ males and females treated with dexamethasone at 4 mg/L. (DOCX) [file pone.0151433.s004.docx]
