# Supplementary material for: Asparaginase Potentiates Glucocorticoid-Induced Osteonecrosis in a Mouse Model
Source: PLoS One. 2016 Mar 11;11(3):e0151433. doi: 10.1371/journal.pone.0151433 (PMC4788417; doi:10.1371/journal.pone.0151433)
Supplement: S1 Methods — (DOCX) [file pone.0151433.s005.docx]

## S1 Methods. Mouse model of dexamethasone-induced osteonecrosis.

We previously showed that BALB/cJ mice had a higher frequency of osteonecrosis with acceptable survival rates compared to BALB/cAnN mice.[[1](#_ENREF_1)] Multiple risk factors of osteonecrosis were evaluated using BALB/cAnNHsd and BALB/cJ mice, including host-related factors such as age and gender, and treatment-related factors such as dexamethasone dose and treatment duration. Without antibiotics, mortality was unacceptable (S2 Fig); all other experiments include antibiotics. Indirect health surveillance was accomplished with ICR sentinel mice by dirty-bedding exchange weekly. The sentinels were tested quarterly, and were found to be free of Sendai virus, mouse parvovirus, minute virus of mice, mouse hepatitis virus, Theiler murine encephalomyelitis virus, epizootic diarrhea of infant mice, pneumonia virus of mice, reovirus, K virus, polyoma virus, *Mycoplasma pulmonis*, lymphocytic choriomeningitis virus, mouse adenovirus, ectromelia virus, and ecto- and endoparasites.

Dexamethasone showed a dose-dependent toxicity in both strains. A high loading dose of 8 mg/L in the first week increased the incidence of osteonecrosis from 23.7% to 40.9% in P28 (postnatal day 28) vendor-derived BALB/cJ mice at 6 weeks, and also significantly reduced the survival (28% vs. 55%, P = 0.025; S2 Fig), compared to those treated with 4 mg/L without a loading dose. Similarly, when the dose of dexamethasone was increased from 4 mg/L to 8 mg/L, the incidence of osteonecrosis increased from 6.7% (1 of 15) to 35% (7 of 20) at 6 weeks of therapy in P24 BALB/cAnNHsd mice. Finally, longer treatment duration seems to slightly increase the incidence of osteonecrosis, especially in P24 BALB/cAnNHsd mice (45% at 8 weeks vs 35% at 6 weeks).

BALB/cJ mice from in-house breeding colonies had different responses to dexamethasone compared with those shipped from a vendor (at 21-24 days of age) including less sepsis-related morbidity (75% vs. 55% survival, P = 0.016; S2 Fig) and a higher incidence of osteonecrosis (49.5% vs. 23.7%, P = 0.025). One hypothesis for the high mortality of vendor-derived mice is the stress caused by transportation and unfamiliar environment. The relationship between stress and disease susceptibility has been demonstrated in many animal models,[[2](#_ENREF_2), [3](#_ENREF_3)] and BALB/c was relatively stress-vulnerable among several strains.[[4-6](#_ENREF_4)] Olfe et al [[7](#_ENREF_7)] also showed that transportation during the juvenile stage caused a long-term stress-induced lymphocytopenia in vendor-derived BALB/c mice compared with those derived from in-house breeding. Another hypothesis is that mice shipped from a vendor vs. bred in-house would have different gastrointestinal flora, and differing microbial sensitivity to prophylactic antibiotics may also have contributed to the difference in survival rates. Lethal gut-derived sepsis was likely caused by resistant *E. faecalis*, which was isolated from enteric cultures from healthy vendor-derived BALB/cJ mice, and blood cultures from moribund vendor-derived BALB/cJ mice after dexamethasone (data not shown). As death from sepsis may be a “competing event” for osteonecrosis, it was necessary to achieve an adequate level of survival to estimate impact of risk factors for osteonecrosis.

Plasma dexamethasone levels were marginally higher in in-house bred BALB/cJ mice than in vendor-derived BALB/cJ (P = 0.04; S3 Fig), consistent with the high incidence of osteonecrosis in in-house bred BALB/cJ mice (S2 Fig). Furthermore, dexamethasone levels were also higher in mice with osteonecrosis than those without osteonecrosis (P = 0.013).

Age has been known to be a critical factor for osteonecrosis. Adolescents (10-20 years old) were at higher risk than children less than 10 years old [[8-17](#_ENREF_8)] and adults over 20 years old.[[10](#_ENREF_10)] It is hypothesized that hormonal and physiologic changes during puberty, and the skeletal maturation, may contribute to their susceptibility to osteonecrosis.[[18](#_ENREF_18)] The impact of age on the development of osteonecrosis was recapitulated in our mouse model. We have determined that the onset of puberty in BALB/c males occurs at postnatal 28 ±1 days (data not shown). Osteonecrosis was mostly observed in mice that started treatment between 4 and 8 weeks of age, with incidence decreasing as the age of treatment onset increased, regardless of dexamethasone dose and treatment duration (data not shown). Earlier start of treatment (postnatal day 24 vs 28) led to a non-significant increase in the incidence of osteonecrosis (0/20 or 0% vs 1/15 or 7% in BALB/cAnN; 9/22 or 41% vs 5/11 or 45% in BALB/cJ). However, earlier start of treatment resulted in significantly lower survival rate of both strains (P = 0.018 for BALB/cAnN and P = 0.026 for BALB/cJ). We hypothesize that starting treatment right before onset of puberty probably maintained plasma dexamethasone at high levels for the whole pubertal period, resulting in an increased incidence of osteonecrosis in mice. However, earlier treatment significantly increased dexamethasone toxicity, and resulted in high mortality of BALB/cJ mice due to sepsis.

There are conflicting clinical data regarding the gender-dependent difference in risk of glucocorticoid-induced osteonecrosis in patients. Some studies reported that girls had higher risk than boys,[[11](#_ENREF_11), [13-15](#_ENREF_13), [17](#_ENREF_17)] but the difference was absent in several other studies.[[8](#_ENREF_8), [10](#_ENREF_10), [16](#_ENREF_16)] The incidence of osteonecrosis in mice, unlike in the patients, was significantly higher among males than females after 6 weeks of treatment with 4 mg/L dexamethasone (48/97 vs 5/78, P < 0.0001; S4B Fig). There were no significant difference between males and females in the survival rates (S4A Fig) and plasma levels of dexamethasone (61.4 nM vs. 69.3 nM in average, P = 0.1; S4C Fig) and corticosterone, suggesting some intrinsic sensitivity to osteonecrosis (e.g. sex hormones, bone or vasculature properties) may predispose male mice to osteonecrosis.

In summary, we report that development of dexamethasone-induced osteonecrosis in mice was affected by multiple factors, including age, gender, dexamethasone dose, treatment duration, substrain and sources of mice. Four-week-old in-house bred BALB/cJ male mice displayed high susceptibility to osteonecrosis with an acceptable survival rate; therefore they were used for the experiments to evaluate the effect of asparaginase on glucocorticoid-induced osteonecrosis.

# References

1. Kawedia JD, Janke L, Funk AJ, Ramsey LB, Liu C, Jenkins D, et al. Substrain-specific differences in survival and osteonecrosis incidence in a mouse model. Comparative medicine. 2012;62(6):466-71. PubMed PMID: 23561879; PubMed Central PMCID: PMC3527750.

2. Sheridan JF, Dobbs C, Brown D, Zwilling B. Psychoneuroimmunology: stress effects on pathogenesis and immunity during infection. Clinical microbiology reviews. 1994;7(2):200-12. PubMed PMID: 8055468; PubMed Central PMCID: PMC358318.

3. Borysenko M, Borysenko J. Stress, behavior, and immunity: animal models and mediating mechanisms. General hospital psychiatry. 1982;4(1):59-67. PubMed PMID: 6281128.

4. Brinks V, van der Mark M, de Kloet R, Oitzl M. Emotion and cognition in high and low stress sensitive mouse strains: a combined neuroendocrine and behavioral study in BALB/c and C57BL/6J mice. Frontiers in behavioral neuroscience. 2007;1:8. doi: 10.3389/neuro.08.008.2007. PubMed PMID: 18958190; PubMed Central PMCID: PMC2525853.

5. Palumbo ML, Zorrilla Zubilete MA, Cremaschi GA, Genaro AM. Different effect of chronic stress on learning and memory in BALB/c and C57BL/6 inbred mice: Involvement of hippocampal NO production and PKC activity. Stress. 2009;12(4):350-61. doi: 10.1080/10253890802506383. PubMed PMID: 19006005.

6. Van Loo PL, Van der Meer E, Kruitwagen CL, Koolhaas JM, Van Zutphen LF, Baumans V. Long-term effects of husbandry procedures on stress-related parameters in male mice of two strains. Laboratory animals. 2004;38(2):169-77. doi: 10.1258/002367704322968858. PubMed PMID: 15070457.

7. Olfe J, Domanska G, Schuett C, Kiank C. Different stress-related phenotypes of BALB/c mice from in-house or vendor: alterations of the sympathetic and HPA axis responsiveness. BMC physiology. 2010;10:2. doi: 10.1186/1472-6793-10-2. PubMed PMID: 20214799; PubMed Central PMCID: PMC2845127.

8. Kawedia JD, Kaste SC, Pei D, Panetta JC, Cai X, Cheng C, et al. Pharmacokinetic, pharmacodynamic, and pharmacogenetic determinants of osteonecrosis in children with acute lymphoblastic leukemia. Blood. 2011;117(8):2340-7. Epub 2010/12/15. doi: 10.1182/blood-2010-10-311969. PubMed PMID: 21148812; PubMed Central PMCID: PMC3062406.

9. Relling MV, Yang W, Das S, Cook EH, Rosner GL, Neel M, et al. Pharmacogenetic risk factors for osteonecrosis of the hip among children with leukemia. Journal of clinical oncology : official journal of the American Society of Clinical Oncology. 2004;22(19):3930-6.

10. Patel B, Richards SM, Rowe JM, Goldstone AH, Fielding AK. High incidence of avascular necrosis in adolescents with acute lymphoblastic leukaemia: a UKALL XII analysis. Leukemia. 2008;22(2):308-12.

11. Mattano LA, Jr., Sather HN, Trigg ME, Nachman JB. Osteonecrosis as a complication of treating acute lymphoblastic leukemia in children: a report from the Children's Cancer Group. Journal of clinical oncology : official journal of the American Society of Clinical Oncology. 2000;18(18):3262-72.

12. Ribeiro RC, Fletcher BD, Kennedy W, Harrison PL, Neel MD, Kaste SC, et al. Magnetic resonance imaging detection of avascular necrosis of the bone in children receiving intensive prednisone therapy for acute lymphoblastic leukemia or non-Hodgkin lymphoma. Leukemia. 2001;15(6):891-7.

13. Mattano LA, Jr., Devidas M, Nachman JB, Sather HN, Hunger SP, Steinherz PG, et al. Effect of alternate-week versus continuous dexamethasone scheduling on the risk of osteonecrosis in paediatric patients with acute lymphoblastic leukaemia: results from the CCG-1961 randomised cohort trial. The lancet oncology. 2012;13(9):906-15. Epub 2012/08/21. doi: S1470-2045(12)70274-7 [pii]

10.1016/S1470-2045(12)70274-7. PubMed PMID: 22901620; PubMed Central PMCID: PMC3448283.

14. Niinimaki RA, Harila-Saari AH, Jartti AE, Seuri RM, Riikonen PV, Paakko EL, et al. High body mass index increases the risk for osteonecrosis in children with acute lymphoblastic leukemia. J ClinOncol. 2007;25(12):1498-504.

15. te Winkel ML, Pieters R, Hop WC, de Groot-Kruseman HA, Lequin MH, van der Sluis IM, et al. Prospective study on incidence, risk factors, and long-term outcome of osteonecrosis in pediatric acute lymphoblastic leukemia. Journal of clinical oncology : official journal of the American Society of Clinical Oncology. 2011;29(31):4143-50. doi: 10.1200/JCO.2011.37.3217. PubMed PMID: 21947829.

16. Burger B, Beier R, Zimmermann M, Beck JD, Reiter A, Schrappe M. Osteonecrosis: a treatment related toxicity in childhood acute lymphoblastic leukemia (ALL)--experiences from trial ALL-BFM 95. PediatrBlood Cancer. 2005;44(3):220-5.

17. Arico M, Boccalatte MF, Silvestri D, Barisone E, Messina C, Chiesa R, et al. Osteonecrosis: An emerging complication of intensive chemotherapy for childhood acute lymphoblastic leukemia. Haematologica. 2003;88(7):747-53.

18. Haajanen J, Saarinen O, Laasonen L, Kuhlback B, Edgren J, Slatis P. Steroid treatment and aseptic necrosis of the femoral head in renal transplant recipients. Transpl P. 1984;16(5):1316-9. Epub 1984/10/01. PubMed PMID: 6385405.
